# The effects of fat consumption on low-density lipoprotein particle size in healthy individuals: a narrative review

**DOI:** 10.1186/s12944-021-01501-0

**Published:** 2021-08-06

**Authors:** Erik Froyen

**Affiliations:** Department of Nutrition and Food Science, Huntley College of Agriculture, California State Polytechnic University, 3801 West Temple Avenue, Pomona, CA 91768 USA

**Keywords:** Low-density lipoprotein size, Fat, Fatty acids, Cardiovascular disease, Human, Clinical trial

## Abstract

Cardiovascular disease (CVD) is the number one contributor to death in the United States and worldwide. A risk factor for CVD is high serum low-density lipoprotein cholesterol (LDL-C) concentrations; however, LDL particles exist in a variety of sizes that may differentially affect the progression of CVD. The small, dense LDL particles, compared to the large, buoyant LDL subclass, are considered to be more atherogenic. It has been suggested that replacing saturated fatty acids with monounsaturated and polyunsaturated fatty acids decreases the risk for CVD. However, certain studies are not in agreement with this recommendation, as saturated fatty acid intake did not increase the risk for CVD, cardiovascular events, and/or mortality. Furthermore, consumption of saturated fat has been demonstrated to increase large, buoyant LDL particles, which may explain, in part, for the differing outcomes regarding fat consumption on CVD risk. Therefore, the objective was to review intervention trials that explored the effects of fat consumption on LDL particle size in healthy individuals. PubMed and Web of Science were utilized during the search process for journal articles. The results of this review provided evidence that fat consumption increases large, buoyant LDL and/or decreases small, dense LDL particles, and therefore, influences CVD risk.

## Introduction

Cardiovascular disease (CVD) (includes heart disease and stroke) is the number one cause of death in the United States [1] and worldwide [2]. A risk factor for CVD is high serum concentrations of low-density lipoprotein cholesterol (LDL-C) [3–6], which may be significantly increased by saturated fatty acids [7]; however, some individuals with heart disease have normal levels of LDL-C [8–10]. Furthermore, LDL particles consist of a variety of sizes which may differentially affect the development of CVD [9, 11, 12]. There are larger, more buoyant LDL particles and smaller, denser LDL particles. These smaller, denser LDL particles are considered to be more atherogenic, and therefore, a significant risk factor for CVD and cardiovascular events [12–23]. LDL subclasses may be characterized by densities and diameters [11, 24–27], as presented in Table 1.

**Table 1:** The properties of LDL subclasses

LDL subclass	Density (g/mL)	Diameter (nm)	Size
1: LDL-I	1.019–1.023	27.2–28.5	Larger
2: LDL-IIa	1.023–1.028	26.5–27.2	Larger
3: LDL-IIb	1.028–1.034	25.6–26.5	Medium
4: LDL-IIIa	1.034–1.041	24.7–25.6	Medium
5: LDL-IIIb	1.041–1.044	24.2–24.7	Smaller
6: LDL-IVa	1.044–1.051	23.3–24.2	Smaller
7: LDL-IVb	1.051–1.063	22.0–23.3	Smaller

*Abbreviations*: *LDL* low-density lipoprotein, *g* grams, *mL* milliliters, *nm* nanometers

The atherogenic properties of small, dense (sd) LDL particles include: increased transport into the arterial wall [15, 28], increased binding to arterial wall proteoglycans [29], increased susceptibility to oxidation, which are subsequently taken up by macrophages as part of the plaque formation process [28, 30], and decreased binding to the LDL receptor [15, 31, 32]. Individuals may be characterized as pattern A (predominance of larger, more buoyant LDL particles) or pattern B (predominance of smaller, denser LDL particles) [16]. The peak particle diameter for pattern A is greater than 25.5 nm, whereas pattern B is characterized by a peak of less than 25.5 nm [16, 33]. Pattern B appears to be more common among men compared to women, which may explain, in part, why males are more at risk for CVD [34–36].

Epidemiological studies have investigated the effects of LDL particle size on CVD risk. In the MESA study, it was discovered that individuals with higher concentrations of sdLDL-C displayed a higher risk for coronary heart disease (CHD) [37]. In addition, as reported in the prospective ARIC investigation, sdLDL-C was associated with an increased risk for CHD [38]. According to the Framingham Offspring Study [39], sdLDL-C was higher in females with CHD compared to controls. In agreement, the Cardiovascular Health Study concluded that small LDL particles were related to CHD in women [40]. These results coincide with the outcomes from the Quebec Cardiovascular Study [41, 42] in which it was demonstrated that sdLDL concentrations increased the risk for heart disease in men. In another study, stroke patients were characterized with having smaller LDL sizes compared to controls; furthermore, sdLDL was a significant predictor of stroke and stroke mortality [43].

Certain studies report that replacing a portion of saturated fat with unsaturated fat – particularly polyunsaturated fat – decreases the risk factors for CVD and total mortality [44–47]. A replacement of 5% energy (E) from saturated fatty acids (SFA) with equivalent E from polyunsaturated fatty acids (PUFA), monounsaturated fatty acids (MUFA), or carbohydrates from whole grains reduced the risk for CHD, according to the Nurses’ Health Study and the Health Professionals Follow Up Study [46–48]. The PREDIMED trial, in which a Mediterranean diet was supplemented with nuts, the participants displayed decreased concentrations of medium-small and very small LDL particles and increased large LDL particles [49]. In the cross-sectional ERA-JUMP study, it was found that serum linoleic acid (the essential omega-6 PUFA) concentrations were inversely associated with small LDL particles [50]. A meta-analysis of randomized controlled trials reported that replacing a portion of saturated fat with polyunsaturated fat decreased CHD events [51].

On the other hand, not all studies and authors are in agreement regarding the replacement of saturated fat with unsaturated fat on decreasing the risk factors for CVD, cardiovascular events, and/or mortality [52–55]. Re-analyses of the Minnesota Coronary Experiment [56] and the Sydney Diet Heart Study [57] found that while replacing saturated fat with linoleic acid decreased serum cholesterol [56], it did not reduce the risk of death from CVD [56, 57]. The PURE prospective cohort study illustrated that intakes of total, saturated, and unsaturated fats were not significantly associated with myocardial infarction risk or CVD mortality. Interestingly, saturated fat consumption lowered the risk of stroke. The types of carbohydrates, however, that were consumed in the baseline diet (whole versus refined grains), as well as the details on fatty acid intakes (trans-fatty acids and vegetable oils) were not identified [58]. A meta-analysis of prospective cohort studies concluded that intake of saturated fat was not associated with an increased CVD risk [59]. Moreover, there was an inverse association between saturated fat consumption and the risk of stroke in a recent meta-analysis of 14 prospective cohort studies [60].

Certain randomized controlled trials have supported the results from epidemiological studies. In a meta-analysis of randomized controlled trials, it was reported that replacing saturated fat with primarily polyunsaturated fat is “unlikely” to lower CVD events or mortality [61]. Replacing saturated fats with polyunsaturated fats was not associated with a lowered risk for secondary prevention of CHD in a meta-analysis of randomized controlled trials [62]. A recent review of 15 randomized controlled trials, Hooper et al. [63] concluded that there is “little or no effect of reducing saturated fat on all-cause mortality or cardiovascular mortality.” It was further stated that “cutting down on saturated fat led to a 17% reduction in the risk for cardiovascular disease (including heart disease and strokes), but had little effect on the risk of dying.”

Altering the diet composition of fat and carbohydrate, for example, low-fat diets, and in turn, high-carbohydrate diets, differentially impacts serum lipid levels. If fat is replaced with carbohydrate, LDL-C is reduced; however, high-density lipoprotein cholesterol (HDL-C) is also reduced and there is an increase in triglycerides (TG) or very-low-density lipoproteins (VLDL) [10, 64–67]. In a recent meta-analysis [68], it was reported that low-carbohydrate diets improve HDL and TG values; however, they also lead to increases in LDL and total cholesterol levels. Another meta-analysis of randomized controlled trials demonstrated that consumption of low-carbohydrate diets generated higher HDL-C and lower TG concentrations compared with low-fat diets [69]. Adhering to a low-carbohydrate, high-fat diet also results in decreased blood glucose and insulin concentrations [70].

As noted, there is controversy regarding whether dietary fatty acid compositions increase the risk for CVD and/or mortality – and if the mechanisms involve LDL modifications – as saturated fat has been demonstrated to increase large, buoyant LDL particles and/or decrease the sdLDL subclass [14, 71, 72]. Additionally, based on a search of the literature, a review article has not been published on this topic. Therefore, the objective is to review intervention trials that explored the effects of fat consumption on LDL particle size in healthy individuals.

## Methods

**Fig. 1:**
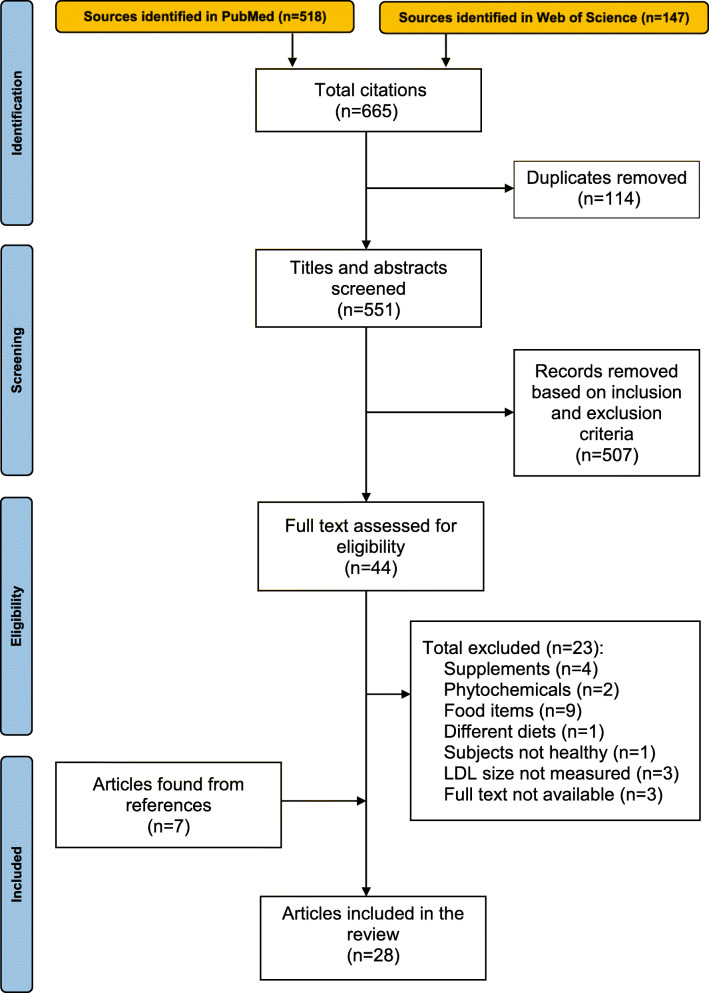
PRISMA flow diagram [73]

The procedure for this narrative review included searching PubMed and Web of Science for peer-reviewed journal articles. The accepted articles on intervention trials that investigated the effects of fat consumption on LDL particle size were published from 1995 to 2021. Overall, the articles used as references ranged from 1978 to 2021. The following search terms were used: low density lipoprotein, small dense LDL, dense LDL, small LDL, LDL subclasses, LDL subfractions, LDL size, LDL classes, LDL fractions, LDL diameter, healthy human clinical trial, healthy human intervention trial, healthy human randomized controlled trial, and combinations thereof. Inclusion criteria included the following: 1) specific fatty acids or saturated, monounsaturated, and polyunsaturated fatty acids consumed in grams (g), g/100 g, or % E were stated; 2) the authors indicated that the participants were “healthy”; 3) the participants had no history of CVD or other chronic diseases; 4) lipid-lowering drugs were not taken; and 5) details on the effects of varying amounts of fatty acids or fat on LDL particle size were reported. There were no restrictions on the length of the studies. The following topics that investigated whether they impacted LDL particle size were excluded: drugs, exercise, calorie-restriction, pregnancy/lactation, fibers, phytochemicals, particular food items, various diets with differing food items, supplements with multiple ingredients, plant sterols and stanols, nutrition education, children (< 18 years), genetic variants, and postprandial studies. The references of the journal articles found via PubMed and Web of Science were also analyzed for additional articles. Using this strategy, 28 journal articles were found (Fig. 1). The journal articles include studies that investigated the effects of individual fatty acids or saturated, monounsaturated, and polyunsaturated fatty acids. Therefore, the collection of journal articles includes studies on the effects of individual fatty acids, as well as saturated, monounsaturated, and polyunsaturated fatty acids, in general, on LDL particle size. As such, both types of studies are reviewed.

It should also be underscored that a variety of techniques may be used to determine LDL particle size [24, 74, 75], such as nondenaturing polyacrylamide gradient gel electrophoresis (PAGGE), analytic or density gradient ultracentrifugation (UC), nuclear magnetic resonance (NMR) spectroscopy [75], and ion mobility (IM) [76]. PAGGE is used to separate LDL particles by size and shape – in addition to determining peak particle diameter [71, 75]. UC typically detects the mass and density (g/mL) of up to four LDL subclasses (LDL-I to LDL-IV) [11, 71, 77]. NMR spectroscopy may be used to measure serum concentrations of LDL subclasses, particle sizes, and numbers [24, 78], while IM detects concentrations of LDL particles based on their size [76, 79]. Thus, the reviewed studies included these techniques, which are highlighted in Table 2. Additionally, the language used in the reviewed studies regarding LDL particle size is summarized in the results column of the table.

## Results

A summary of the results for the reviewed journal articles is provided in Table 2. The results in the table focus on the effects of fatty acid or fat consumption on LDL particle size. The effects of the treatments are indicated above the respective treatments, which are stated in parentheses. Following the table, the results are organized according to the effects of dietary fatty acid or fat compositions on LDL particle size.

**Table 2:** Intervention studies on the effects of fat intake on LDL particle size

Author (Year)	Study/Method	Subjects	Age	Duration	Treatment	Results
Campos et al. (1995) [80]	Randomized Crossover PAGGE UC	43 males	Mean, 50 years (SD, ± 11)	12 weeks	Each diet was consumed for 6 wk. **Low-fat diet:** 24.2% E fat (6% E SFA, 11.6% E MUFA, 4.3% E PUFA), 58.8% E CHO, 16.8% E PRO. **High-fat diet:** 45.2% E fat (18.1% E SFA, 12.4% E MUFA, 11.8% E PUFA), 39.2% E CHO, 16.3% E PRO. Calories, cholesterol, fiber, and P:S were kept constant.	↓LDL-C (Low-fat diet) ↑mean peak LDL diameter ↓LDL peak density ↑large, buoyant LDL particle mass (LDL I and LDL II) ↓sdLDL particle mass (LDL III and LDL IV) (High-fat diet)
Krauss et al. (1995) [77]	Randomized Crossover PAGGE UC	105 males	28 to 79 years mean, 48.9 (SD, ± 11.1)	12 weeks	Each diet was consumed for 6 wk. **Low-fat diet:** 23.9% E fat (5.4% E SFA, 12.3% E MUFA, 4% E PUFA), 60% E CHO, 16.1% E PRO. **High-fat diet:** 46% E fat (18.3% E SFA, 12.4% E MUFA, 12.5% E PUFA), 38.6% E CHO, 16.2% E PRO.	↓LDL-C (Low-fat diet) ↑large, buoyant LDL particle mass (LDL I and LDL II) ↓sdLDL particle mass (LDL III and LDL IV) (High-fat diet) Thirty-six subjects (about one-third) switched from pattern A (or intermediate pattern) to pattern B by following the low-fat diet.
Carmena et al. (1996) [81]	Intervention PAGGE	18 males	30 to 69 years mean, 57.1 (SD, ± 17.2)	6 weeks	The SFO diet was consumed for 3 wk., followed by 3 wk. on the OO diet. **Sunflower seed oil (SFO) diet:** 31% E fat (6.8% E SFA, 10.9% E MUFA, 13.3% E PUFA), 48% E CHO, 11% E PRO. **Olive oil (OO) diet:** 30.5% E fat (6.9% E SFA, 21.6% E MUFA, 4.7% E PUFA), 48% E CHO, 11% E PRO. Vitamin E, beta-carotene, and vitamin C were significantly higher in the SFO diet.	↓LDL-C ↑LDL size (SFO diet compared to OO diet)
Kasim-Karakas et al. (1997) [82]	Intervention PAGGE	14 females	Mean, 61 years (SD, ± 11)	4 months	Consumption of a “habitual diet”, followed by the intakes of a 31% fat diet for 4 wk., followed by the 24% fat diet for 6 wk., then the 14% fat diet for 6 wk. **Habitual diet:** 33% E fat (10% E SFA, 13% E MUFA, 8% E PUFA), 51% E CHO, 71 g PRO. **31% fat diet:** 31% E fat (10% E SFA, 12% E MUFA, 9% E PUFA), 53% E CHO, 17% E PRO. **24% fat diet:** 23% E fat (6% E SFA, 10% E MUFA, 7% E PUFA), 60% E CHO, 18% E PRO. **14% fat diet:** 14% E fat (3% E SFA, 7% E MUFA, 4% E PUFA), 67% E CHO, 19% E PRO.	↓LDL-C (14% fat diet) ↔LDL particle size (all diets compared)
Clifton et al. (1998) [26]	Randomized Double-blind PAGGE	54 males 51 females	Males: 30–66 years mean, 50 years (SD, ± 7.5) Females: 23–76 years mean, 51.1 years (SD, ± 9.5)	8 weeks	Consumption of a “self-selected” low fat baseline diet for 2 wk., followed by the addition of a fat-containing (high fat phase) or fat free (low fat phase) liquid supplement to the baseline diet for 3 wk. each. **Low fat baseline diet:** 26.2% E fat (9% E SFA, 9% E MUFA, 5.4% E PUFA), 51.1% E CHO, 19.7% E PRO. **Low fat phase:** 20.7% E fat (7.5% E SFA, 7.1% E MUFA, 4.3% E PUFA), 59.3% E CHO, 20% E PRO. **High fat phase:** 35.7% E fat (14.9% E SFA, 12.1% E MUFA, 3.7% E PUFA), 46.3% E CHO, 18% E PRO. The high fat phase had significantly higher cholesterol (748 mg) compared to the low fat phase (182 mg)	↑LDL-C (high fat phase compared to low fat phase) ↑smaller LDL particles (men compared to women, in both low fat and high fat phases) ↔LDL particle size (low fat compared to high fat phase)
Dreon et al. (1998) [71]	Randomized Crossover PAGGE UC	103 males	28–79 years mean, 48.9 years (SD, ± 11.1)	12 weeks	Consumption of each experimental diet for 6 wk. **Low-fat diet:** 24.2% E fat, 5.9% E SFA (0.1% E lauric acid, 0.3% E myristic acid, 3.7% E PA, 1.5% E SA), 11.8% E MUFA (11.7% E OA), 4.2% E PUFA (0.1% E AA, 0.3% E ALA, 3.9% E LA), 59% E CHO, 16.6% E PRO. **High-fat diet:** 45.5% E fat, 18.4% E SFA (0.5% E lauric acid, 2.3% E myristic acid, 4% E SA, 9% E PA), 12.5% E MUFA (11.7% E OA), 11.8% E PUFA (0.6% E ALA, 10.8% E LA), 38.8% E CHO, 16.3% E PRO. Significant differences in reported intakes of cholesterol, P:S, and fiber.	↑LDL-C (High-fat diet) ↔plasma lipoproteins [SA, MUFA (and OA), PUFA (and LA)] ↑large LDL particle mass ↑LDL peak particle diameter (High-fat diet, high SFA, myristic and palmitic acids) ↓sdLDL mass (High-fat diet; total SFA; myristic acid) Dietary protein, carbohydrate, cholesterol, P:S, and fiber were not associated with plasma lipoproteins.
Lagrost et al. (1999) [83]	Randomized PAGGE	32 total 14 males 18 females	20–60 years (mean, 41 years)	23 weeks	Three different diets were consumed for 6 wk. each, with 2 to 3 wk. washout periods. **Lauric acid diet:** 41.5% E fat, 22.2% E SFA (10.6% E lauric acid, 4.2% E myristic acid, 5.9% E PA), 11% E MUFA (10.3% E OA), 4.6% E PUFA (4.4% E LA), 43.9% E CHO, 14.3% E PRO. **Palmitic acid diet:** 41% E fat, 18.9% E SFA (13.2% E PA, 2.2% E myristic acid, 1.9% E lauric acid), 11.4% E MUFA (10.6% E OA), 4.6% E PUFA (4.4% E LA), 44.7% E CHO, 14.3% E PRO. **Oleic acid diet:** 41.8% E fat, 11.8% E SFA (5.7% E PA, 2.5% E lauric acid, 1.9% E myristic acid), 19.9% E MUFA (19% E OA), 5.4% E PUFA (5.2% E LA), 44.3% E CHO, 14% E PRO. Nutrient compositions were similar for each diet, except about 8.5% E was supplied by lauric (+ 2.2% E myristic acid), palmitic, or oleic acids.	↓LDL-C (Oleic acid diet compared to lauric acid and palmitic acid diets). There were no significant differences between lauric acid and palmitic acid diets. ↔LDL particle mean size (all diets compared)
Dreon et al. (1999) [84]	Randomized Crossover PAGGE UC	38 males	32–71 years mean, 52.5 years (SD, ± 12.1)	20 days	Participants displayed phenotype A by following both a low- and high-fat diet for 4–6 wk. in a previous study. Consumption of their usual diet and very-low-fat diet for 10 d each. **Usual diet:** 31.8% E fat (10.8% E SFA, 11.8% E MUFA, 6.9% E PUFA), 52.1% E CHO, 14% E PRO. **10%-Fat diet:** 10.4% E fat (2.7% E SFA, 3.7% E MUFA, 2.6% E PUFA), 75.7% E CHO, 14.5% E PRO.	↓LDL-C ↓mass larger LDL-I ↑mass smaller LDL-III and LDL-IV subfractions ↓LDL particle size ↓LDL peak diameter (10%-Fat diet compared to usual diet) Twelve individuals (about one-third) converted to phenotype B, whereas 26 remained phenotype A.
Pedersen et al. (2000) [27]	Randomized Double-blind Crossover UC	18 males	20–28 years (mean, 24 years)	Up to 33 weeks	Three identical diets were consumed for 3 wk. each (5–12 wk. washout periods), except that 19% E was from either extra virgin olive oil, physically refined rapeseed oil, or chemically refined sunflower oil. **Olive oil (OO) diet:** 35% E fat (11% E SFA, 21% E MUFA, 3% E PUFA), 53% E CHO, 12% E PRO. **Rapeseed oil (RO) diet:** 35% E fat (9% E SFA, 18% E MUFA, 7% E PUFA), 52% E CHO, 13% E PRO. **Sunflower oil (SO) diet:** 35% E fat (10% E SFA, 9% E MUFA, 15% E PUFA), 53% E CHO, 12% E PRO. OO diet contained significantly more squalene and less campesterol and sitosterol compared to RO and SO diets.	↓LDL-C (RO and SO diets compared to OO diet) ↔LDL subfraction average size (all diets compared) ↑number of larger and medium-sized LDL subfractions (LDL-1 to LDL-3) (OO diet compared to RO and SO diets) ↑number of medium-sized and sdLDL subfractions (LDL-4 to LDL-5) (OO diet compared to RO diet) ↔number of smallest, dense LDL particles (LDL-6) (all diets compared)
Kratz et al. (2002) [85]	Randomized Parallel PAGGE	56 total 30 males 26 females	18 to 43 years (69 initial participants) mean, 25.8 years (SD, ± 5.5)	6 weeks	Baseline diet rich in SFA was consumed for 2 wk., followed by participants assigned to one of three treatment diets for 4 wk. **Baseline diet:** 38% E fat (19% E SFA, 11.2% E MUFA, 5.2% E n-6 PUFA, 0.4% E n-3 PUFA, 45.1% E CHO, 16.9% E PRO. **Refined olive oil diet:** 38.7% E fat (10.7% E SFA, 23.2% E MUFA, 3% E n-6 PUFA, 0.4% E n-3 PUFA, 47% E CHO, 14.3% E PRO. **Rapeseed oil diet:** 38.4% E fat (9.1% E SFA, 19.1% E MUFA, 6.5% E n-6 PUFA, 2.5% E n-3 PUFA, 47.3% E CHO, 14.3% E PRO. **Sunflower oil diet:** 38.3% E fat (10% E SFA, 8.7% E MUFA, 18.2% E n-6 PUFA, 0.3% E n-3 PUFA, 47.6% E CHO, 14.2% E PRO. The diets were identical, save for fatty acid composition.	↓LDL size ↓LDL peak particle diameter (all 3 diets compared to baseline diet) ↔LDL size (all 3 treatment diets compared)
Sharman et al. (2002) [33]	Intervention PAGE (nongradient)	20 males	Ketogenic diet: mean, 36.7 years (SD, ± 11.6) Control diet: mean, 35 years (SD, ± 13)	6 weeks	Twelve subjects switched from their usual dietary pattern to a ketogenic diet, whereas 8 subjects continued their usual dietary pattern (controls) for 6 wk. **Ketogenic diet:** 61% E fat (25% E SFA, 25% E MUFA, 11% E PUFA), 8% E CHO, 30% E PRO. **Habitual diet:** 25% E fat (12% E SFA, 9% E MUFA, 4% E PUFA), 59% E CHO, 15% E PRO. All nutrients were significantly different between diets, except for energy and alcohol consumption.	↔LDL-C (both diets after 6 wk) ↑LDL peak particle diameter (ketogenic diet after 3 wk) ↑LDL-1 percentage (ketogenic diet) All 7 initial pattern A subjects remained pattern A after the ketogenic diet (no significant changes in percentages of any LDL subclasses, or the mean and peak LDL particle size). Most initial pattern B subjects (3 out of 5) changed to pattern A after the ketogenic diet.
Rivellese et al. (2003) [86]	Randomized PAGGE UC	162 total 86 males 76 females	30–65 years SFA diet: mean, 48 years (SD, ± 8) (n-3 group) and mean, 49 years (SD, ± 7) (placebo) MUFA diet: mean, 49 years (SD, ± 7) (n-3 group and placebo)	90 days	Consumption of a diet high in SFA or MUFA, followed by a second random assignment to capsule supplements of fish oil (3.6 g n-3 FA, containing 2.4 g EPA and DHA) or placebo capsules (with same amount of olive oil). The test period was preceded by a 2 wk. “stabilisation period” on their “habitual” diets and placebo capsules. **SFA diet:** 37.1% E fat (17.6% E SFA, 13.1% E MUFA, 4.7% E PUFA), 44.1% E CHO, 15.2% E PRO. **MUFA diet:** 37.1% E fat (9.6% E SFA, 21.2% E MUFA, 4.6% E PUFA), 45.9% E CHO, 14.8% E PRO.	↑LDL-C (SFA diet compared to MUFA diet) ↑LDL-C (n-3 supplementation in both diets) ↔LDL size (all diets compared)
Archer et al. (2003) [87]	Randomized PAGGE	65 males	Mean, 37.5 years (SD, ± 11.2)	6–7 weeks	Subjects consumed one of the diets for 6–7 wk. in an ad libitum manner. **Low fat, high CHO diet:** 25.8% E fat (6% E SFA, 13.3% E MUFA, 5.1% E PUFA. 58.3% E CHO, 15.9% E PRO. **High MUFA diet:** 40.1% E fat (8.2% E SFA, 22.5% E MUFA, 7.6% E PUFA, 44.7% E CHO, 15.2% E PRO.	↓LDL-C (both diets; no significant difference between diets) ↓LDL peak particle diameter (High CHO diet; in subjects with large LDL peak particle diameters at baseline) ↑percentage of small LDL particles (High CHO diet; no significant difference between diets)
Smith et al. (2003) [88]	Randomized Single-blind Parallel UC	51 total 26 males 25 females	18–28 years Moderate MUFA diet: Males: mean, 21 years (SD, ± 3) Females: mean, 20 (SD, ± 1) High MUFA diet: Males: mean, 20 years (SD, ± 2) Females: mean, 20 years (SD, ± 2)	24 weeks	Consumption of a SFA-rich reference diet for 8 wk., followed by either a moderate- or high-MUFA diet for 16 wk. **SFA reference diet (one for each MUFA diet):** 39.8% E/37.7% E fat (15.4% E/14.5% E SFA, 12.5% E/11.9% E MUFA, 7.3% E/6.7% E PUFA, 47.9% E/50% E CHO, 10.5% E/10.7% E PRO **Moderate-MUFA diet:** 39.7% E fat (12.1% E SFA, 15.1% E MUFA, 7.2% E PUFA), 47.7% E CHO, 11.2% E PRO **High-MUFA diet:** 37.1% E fat (9.7% E SFA, 16.6% E MUFA, 6.9% E PUFA, 50.3% E CHO, 11% E PRO MUFA intakes were not significantly different between the two MUFA diets. MUFA intakes were significantly higher and SFA intakes were significantly lower than the reference diets.	↓LDL-C (moderate- and high-MUFA diets compared to baseline, after SFA reference diet) ↑LDL-1 percentage (moderate-MUFA diet compared to SFA reference diet) ↔proportions of LDL subfractions (between each diet)
Volek et al. (2003) [89]	Randomized Crossover PAGE (nongradient)	10 females	Mean, 26.3 years (SD, ± 6.1)	12 weeks	Each diet was consumed for 4 wk., with a 4 wk. break between diets. **Very low CHO diet:** 60% E, 118 g fat (41 g SFA, 35 g MUFA, 20 g PUFA), 10% E CHO (43 g), 29% E PRO (128 g). **Low fat diet:** 19% E, 34 g fat (10 g SFA, 9 g MUFA, 6 g PUFA), 62% E CHO (249 g), 17% E PRO (68 g).	↑LDL-C (very low CHO diet compared to baseline and low fat diet) ↔relative percentages or concentrations of LDL subclasses (after consumption of each diet) Three of ten participants with pattern B displayed larger peak LDL size after following the very low CHO diet.
Goyens et al. (2005) [78]	Randomized Double-blind Parallel NMR	54 total 21 males 33 females 29 total (NMR analyses) 14 males 15 females	Males: mean, 52.6 years (SD, ± 13.7) Females: mean, 47.7 years (SD, ± 11.1)	10 weeks	A 4 wk. period, followed by consumption of one of the following diets for 6 wk. **Control diet:** 33.5% E fat (11.6% E SFA, 12.8% E MUFA, 8% E PUFA, 7.3% E LA and 0.4% E ALA), 50.5% E CHO, 14.5% E PRO. **Low-LA diet:** 34% E fat (12.4% E SFA, 16.9% E MUFA, 3.7% E PUFA, 3% E LA, 0.4% E ALA, 49.8% E CHO, 14.9% E PRO. **High-ALA diet:** 32.6% E fat (10.4% E SFA, 12.6% E MUFA, 8.6% E PUFA, 7.1% E LA, 1.1% E ALA, 50.4% E CHO, 15.5% E PRO.	↓LDL-C (High-ALA diet compared to control diet) ↔mean LDL particle size (all groups compared)
Thijssen et al. (2005) [90]	Randomized Crossover NMR	45 total 18 males 27 females 22 total (NMR analyses) 9 males 13 females	28–66 years mean, 51 years (SD, ± 10)	17 weeks	Consumption of each diet for 5 wk., with a washout period of ≥1 wk. between diets. **Stearic acid diet:** 38.2% E fat, 18% E SFA (7.7% E SA), 12.9% E MUFA (6.8% E OA), 4.7% E PUFA (2.1% E LA, 0.2% E ALA), 45.8% E CHO, 14% E PRO. **Oleic acid diet:** 37.7% E fat, 11% E SFA (1.2% E SA), 19.1% E MUFA (13.1% E OA), 5% E PUFA (2.4% E LA, 0.2% E ALA), 46.3% E CHO, 14% E PRO. **Linoleic acid diet:** 38% E fat, 11.2% E SFA (1.2% E SA), 12.5% E MUFA (6.6% E OA), 11.8% E PUFA (9.3% E LA, 0.2% E ALA), 46.3% E CHO, 13.8% E PRO. The diets did not differ, save for the difference of 7% E from SA, OA, or LA.	↔LDL-C ↔LDL particle size and subclass concentrations (all 3 diets compared)
Faghihnia et al. (2010) [91]	Randomized Crossover PAGGE UC	63 total 61 males 2 females	At least 20 years mean, 47.9 years (SD, ± 11.2)	8 weeks	Each diet was consumed for 4 wk. **High-fat low-carbohydrate diet:** 40% E fat (13% E SFA, 11% E MUFA, 14% E PUFA), 45% E CHO, 15% E PRO. **Low-fat high-carbohydrate diet:** 20% E fat (5% E SFA, 10% E MUFA, 5% E PUFA), 65% E CHO, 15% E PRO. There were no differences in cholesterol and simple:complex CHO ratios.	↓LDL-C ↓large and medium LDL particle concentrations ↑small and very small LDL particle concentrations ↓mean LDL peak particle diameter (Low-fat high-carbohydrate diet compared with the high-fat low-carbohydrate diet)
Egert et al. (2011) [92]	Randomized Parallel PAGGE	37 total 12 males 25 females	18–34 years mean, 22.6 years (SD, ± 4.2)	6 weeks	Consumption of a 2 wk. wash-in SFA-rich diet followed by consumption of one of the treatment diets for 4 wk. **Wash-in SFA-rich diet:** 40.8% E fat (18.1% E SFA, 13.1% E MUFA, 6.6% E n-6 PUFA, 1.1% E n-3 PUFA), 42.6% E CHO, 15.7% E PRO. **Low-fat diet (MUFA-rich):** 28.7% E fat (7.2% E SFA, 13.9% E MUFA, 5.3% E n-6 PUFA, 0.9% E n-3 PUFA), 54.4% E CHO, 15.6% E PRO. **High-fat diet (MUFA-rich):** 40.2% E fat (9.9% E SFA, 19.8% E MUFA, 7% E n-6 PUFA, 1.6% E n-3 PUFA), 43.1% E CHO, 15.6% E PRO. Both diets were isocaloric, rich in MUFA, with similar FA, CHO, cholesterol, fiber, and antioxidant proportions.	↓LDL-C ↓LDL size of the major fraction (both diets compared to the wash-in SFA-rich diet; no significant difference between treatment diets)
Mangravite et al. (2011) [79]	Randomized Crossover IM	40 males	Mean, 45 years (SD, ± 15)	13 weeks	Consumption of a baseline diet for 3 wk., followed by intakes of two intervention diets for 3 wk. each. There were 2 wk. washout periods after the baseline diet and between intervention diets. **Baseline diet:** 38% E fat (15% E SFA, 15% E MUFA, 6% E PUFA), 50% E CHO, 13% E PRO (no beef protein). **Lower carbohydrate, high-saturated fat (LCHSF) diet:** 38% E fat (15% E SFA, 15% E MUFA, 5% E PUFA), 31% E CHO, 31% E PRO (10% E beef protein). **Lower carbohydrate, low-saturated fat (LCLSF) diet:** 38% E fat (8% E SFA, 21% E MUFA, 6% E PUFA), 31% E CHO, 32% E PRO (11% E beef protein).	↓LDL-C ↓total LDL ↓medium LDL concentrations (LCLSF diet compared to LCHSF and baseline diets) ↓small LDL concentrations (LCLSF diet compared to LCHSF diet) ↔large LDL ↔very small LDL ↔LDL peak diameter ↔LDL subclass phenotype (all diets compared)
Faghihnia et al. (2012) [93]	Randomized Crossover UC	14 males	24–67 years mean, 44.5 years (SD, ± 14.4)	11 weeks	Consumption of a baseline diet for 3 wk., followed by intakes of two experimental diets for 3 wk. each. There was a 2 wk. washout period between experimental diets. **Baseline diet:** 38% E fat (15% E SFA, 15% E MUFA, 6% E PUFA), 50% E CHO, 13% E PRO. **Low CHO, high SFA diet:** 38% E fat (15% E SFA, 15% E MUFA, 5% E PUFA), 31% E CHO, 31% E PRO (10% E beef protein). **Low CHO, low SFA diet:** 38% E fat (8% E SFA, 21% E MUFA, 6% E PUFA), 31% E CHO, 31% E PRO (11% E beef protein).	↓LDL-C (low CHO, low SFA diet compared to low CHO, high SFA diet) ↑LDL total mass concentration ↑LDL subclass I (large), II (medium), and III (small) mass concentrations (low CHO, high SFA diet compared to low CHO, low SFA diet) ↔LDL subclass IV (very small) (compared to each diet)
Guay et al. (2012) [94]	Randomized Double-blind Crossover PAGGE	12 males	18 to 50 years mean, 27.1 years (SD, ± 3.9)	2 weeks plus 6 days	Consumption of two experimental diets for 3 d each, separated by a 2 wk. washout period. **Low fat diet:** 25% E fat (6% E SFA, 12% E MUFA, 4.9% E PUFA), 61.8% E CHO, 15% E PRO. **High fat diet:** 37% E fat (15% E SFA, 12.7% E MUFA, 4.3% E PUFA), 49.8% E CHO, 15% E PRO. The experimental diets consisted of the same calories, proteins, fiber, MUFA, and PUFA.	↑LDL-C ↑LDL particle size ↔LDL peak particle diameter ↑percentage of large (not significant) and medium LDL particles ↓percentage of small LDL particles (High fat diet compared with low fat diet)
Wang et al. (2015) [95]	Randomized Crossover NMR	45 total 27 males 18 females	21–70 years mean, 45 years (SD, ± 13.3)	14 weeks	A 2 wk. intake of an average American diet, followed by dietary treatments for 5 wk. each. There was a 2 wk. “compliance break” between treatments. **Average American diet (AAD):** 34% E fat (13% E SFA, 12% E MUFA, 7% E PUFA), 51% E CHO, 16% E PRO. **Lower-fat diet (LF):** 24% E fat (7% E SFA, 11% E MUFA, 6% E PUFA), 59% E CHO, 16–17% E PRO. **Moderate-fat diet (MF):** 34% E fat (6% E SFA, 17% E MUFA, 9% E PUFA), 49% E CHO, 16–17% E PRO. Diets were designed to meet calorie needs.	↓LDL-C ↓large LDL particle number ↓mean LDL particle size (LF and MF compared to AAD; no significant difference between LF and MF) ↓total LDL particle number (MF compared to LF; no significant difference compared to AAD) ↑small LDL particle number (LF and MF compared to AAD; there was also a significant increase with LF compared to MF)
Dias et al. (2017) [96]	Randomized Parallel NMR	26 total 11 males 15 females	21–65 years (29 subjects recruited) SFA-rich diet: median, 32 years n-6 PUFA-rich diet: median, 28 years	4 weeks plus 10 days	Consumption of 4 × 1 g fish oil capsules (100 mg EPA and 500 mg DHA each) for 4 wk., followed by one of the treatment diets for 10 d while consuming the fish oil capsules. **SFA + LC n-3 PUFA diet:** 38.8% E fat (50.4 g SFA/100 g, 34.6 g MUFA/100 g, 13.5 g PUFA/100 g, 9.1 g LA/100 g, 4 g LC n-3 PUFA/100 g), 37.6% E CHO, 17.8% E PRO. **n-6 PUFA + LC n-3 PUFA diet:** 38.6% E fat (25.4 g SFA/100 g, 32.3 g MUFA/100 g, 39.1 g PUFA/100 g, 34.5 g LA/100 g, 4.6 g LC n-3 PUFA/100 g), 34% E CHO, 21% E PRO.	↓LDL-C ↓total LDL particle concentration ↓very large, medium-large, and small LDL particle concentrations (n-6 PUFA + LC n-3 PUFA diet compared to SFA + LC n-3 PUFA diet)
Dias et al. (2017) [97]	Randomized Parallel NMR	26 total 6 males 20 females	18–65 years	6 weeks	Diets were consumed for 6 wk. The diets contained 400 mg EPA and 2000 mg DHA. **SFA-rich diet:** 40.9% E fat (18.9% E SFA, 13.8% E MUFA, 4.4% E PUFA, 2.9% E LA, 1.13% E n-3 PUFA), 38.1% E CHO, 16.6% E PRO. **n-6 PUFA-rich diet:** 42.4% E fat (12.6% E SFA, 13.2% E MUFA, 14.4% E PUFA, 12.7% E LA, 1% E n-3 PUFA), 41.6% E CHO, 18.1% E PRO.	↔LDL-C ↔LDL particle size concentrations (between diets)
Ulven et al. (2019) [98]	Randomized Double-blind NMR	99 total Control diet: 52 total 21 males 31 females Exp. diet: 47 total 20 males 27 females	25–70 years Control diet: mean, 55.2 years (SD, ± 9.8) Exp. diet: mean, 53.6 years (SD, ± 9.7)	10 weeks	A 2 wk. duration which consisted of the control food items, followed by the consumption of 1 of 2 intervention diets for 8 wk. **Control diet:** 42.8% E fat (18% E SFA, 15.4% E MUFA, 5.6% E PUFA), 36.6% E CHO, 15% E PRO. **Experimental diet:** 42.9% E fat (11.5% E SFA, 15.7% E MUFA, 12% E PUFA), 34.2% E CHO, 16.5% E PRO. There was a 6.5% E lower SFA and a 6.4% E higher PUFA in the experimental diet. PRO, CHO, and fiber intakes were also significantly different.	↓LDL-C ↓Large, medium and small LDL particle concentrations (Experimental diet compared to control diet)
Bergeron et al. (2019) [99]	Randomized Parallel (high or low SFA arm) Crossover IM	113 total High-SFA arm: 62 total 27 males 35 females Low-SFA arm: 51 total 17 males 34 females	21–65 years High-SFA arm: mean, 45 years (SD, ± 12) Low-SFA arm: mean, 42 years (SD, ± 13)	Up to 28 weeks	A 2 wk. baseline diet, followed by random assignment to a low-SFA (~ 7% E) or high-SFA (~ 14% E) arm. Within each SFA arm, 3 experimental diets were consumed for 4 wk. each, with a 2–7 wk. washout period between experimental diets. **High-SFA arm:** **Red meat diet:** 35% E fat (13% E SFA, 12% E MUFA, 5% E PUFA), 41% E CHO, 24% E PRO (11.5% E red meat). **White meat diet:** 34% E fat (14% E SFA, 13% E MUFA, 5% E PUFA), 42% E CHO, 24% E PRO (11.5% E white meat). **Nonmeat diet:** 35% E fat (14% E SFA, 12% E MUFA, 6% E PUFA), 41% E CHO, 24% E PRO (15.4% E vegetable protein). **Low-SFA arm:** **Red meat diet:** 35% E fat (8% E SFA, 21% E MUFA, 5% E PUFA), 39% E CHO, 26% E PRO (12.5% E red meat). **White meat diet:** 31% E fat (7% E SFA, 18% E MUFA, 6% E PUFA), 46% E CHO, 23% E PRO (11% E white meat). **Nonmeat diet:** 34% E fat (7% E SFA, 20% E MUFA, 5% E PUFA), 41% E CHO, 25% E PRO (16% E vegetable protein).	↑LDL-C ↑large LDL particle concentrations (High SFA compared with low SFA, independent of protein source) ↔small- and medium-sized LDL particle concentrations (High SFA intake compared with low SFA intake)
Buren et al. (2021) [100]	Randomized Crossover PAGGE	17 females	19–27 years median, 23.8 years	23 weeks	Each diet was consumed for 4 wk., separated by a 15 wk. washout period. **Ketogenic low-carbohydrate high-fat (LCHF) diet:** 77% E fat (33% E SFA), 4% E CHO (not exceeding 25 g, excluding fiber), 19% E PRO. **Control diet:** 33% E fat, 44% E CHO, 19% E PRO.	↑LDL-C ↑sdLDL-C ↑large,buoyant LDL-C (LCHF diet compared to control diet)

*Abbreviations*: *AA* arachidonic acid, *ALA* alpha-linolenic acid, *CHO* carbohydrate, *d* days, *DHA* docosahexaenoic acid, *E* energy, *EPA* eicosapentaenoic acid, *FA* fatty acids, *g* grams, *IM* ion mobility, *LA* linoleic acid, *LC* long chain, *LDL-C* low-density lipoprotein cholesterol, *MUFA* monounsaturated fatty acids, *NMR* nuclear magnetic resonance, *OA* oleic acid, *PA* palmitic acid, *PAGGE* polyacrylamide gradient gel electrophoresis, *PRO* protein, P:S, ratio of polyunsaturated to saturated fatty acids, *PUFA* polyunsaturated fatty acids, *SA* stearic acid, *SD* standard deviation, *sdLDL* small, dense low-density lipoprotein, *SFA* saturated fatty acids, *UC* ultracentrifugation, *wk.* weeks, ↑, increase; ↓, decrease; ↔, no significant difference between groups

### The effects of low-fat and high-fat diets on LDL particle size

In a study by Campos et al. [80], it was demonstrated that the consumption of a high-fat (45.2% E) diet increased large, buoyant LDL particles and reduced sdLDL particles compared to a low-fat (24.2% E) diet. These results are in agreement with similar studies in which the participants consumed a high-fat diet for 6 weeks (wk) [71, 77]. Interestingly, in the investigation by Krauss and Dreon [77], about one-third of pattern A (or intermediate pattern) individuals converted to pattern B by following the low-fat (23.9% E) diet. In the study by Dreon et al. [71], intakes of the high-fat (45.5% E) diet, high SFA (18.4% E), myristic acid (2.3% E), and palmitic acid (9% E) increased large LDL particle mass compared to the low-fat (24.2% E) diet. Additionally, the intake of the high-fat diet, SFA, and myristic acid decreased sdLDL. Interestingly, the consumption of stearic acid (a SFA), MUFA (including oleic acid), and PUFA (including linoleic acid) did not affect plasma lipoproteins.

In agreement with the aforementioned studies, consumption of a very-low-fat (10.4% E) diet for 10 days (d) decreased large LDL and increased smaller LDL fractions compared to a higher fat (31.8% E) diet. Furthermore, about one-third of participants switched to pattern B after following the very-low fat diet [84]. The consumption of a low-fat, high-carbohydrate (CHO) diet (20% E fat and 65% E CHO) for 4 wk. decreased large and medium LDL particle concentrations, while increasing small and very small LDL particle concentrations compared to the high-fat, low-carbohydrate diet (40% E fat and 45% E CHO) [91]. The percentage of small LDL particles decreased while the percentage of medium LDL particles increased after following a high-fat diet (37% E fat, 15% E SFA, and 49.8% E CHO) for 3 d versus a low-fat diet (25% E fat, 6% E SFA, and 61.8% E CHO) [94].

Contrary to these outcomes, an investigation in which post-menopausal females consumed a higher fat (31% E) diet, followed by intakes of lower fat (24% E and 14% E) diets in a step-wise manner, showed no significant differences in LDL size [82]. Another study found no changes in LDL particle size after male and female participants consumed a high-fat diet (35.7% E fat and 14.9% E SFA) for 3 wk. compared to a low-fat diet (20.7% E fat and 7.5% E SFA); however, male participants had higher concentrations of sdLDL particles compared to females [26]. Ad libitum consumption of a low-fat, high-carbohydrate diet (25.8% E fat, 6% E SFA, and 58.3% E CHO) for ~ 7 wk. significantly decreased LDL peak particle diameter (in subjects with large diameters at baseline) and increased the percentage of small LDL particles; however, there were no significant differences compared with the high MUFA diet (40.1% E fat, 8.2% E SFA, and 44.7% E CHO) [87].

### The effects of higher or mixed fat diets on LDL particle size

A study by Lagrost et al. [83] demonstrated that consumption of higher fat (~ 41% E) diets rich in lauric acid (a SFA) (10.6% E), palmitic acid (13.2% E), or oleic acid (19% E) for 6 wk. each resulted in no significant differences in mean LDL size. Consistent with this study, the intakes of a low linoleic acid diet [34% E fat, 3% E linoleic acid, and 0.4% E alpha-linolenic acid (the essential omega-3 fatty acid)] and a high alpha-linolenic acid diet (32.6% E fat, 7.1% E linoleic acid, and 1.1% E alpha-linolenic acid) for 6 wk. did not affect mean LDL particle size compared to the control diet (33.5% E fat, 7.3% E linoleic acid and 0.4% E alpha-linolenic acid) [78]. These results coincide with the consumption of three diets (~ 38% E fat) rich in stearic acid (7.7% E), oleic acid (13.1% E), or linoleic acid (9.3% E) for 5 wk. each in which there were no significant differences in LDL particle size and concentrations [90]. The consumption of diets containing 37.1% E fat, one rich in SFA (17.6% E) and one rich in MUFA (21.2% E) [both supplemented with the omega-3 fatty acids eicosapentaenoic acid (EPA) and docosahexaenoic acid (DHA)], also did not significantly differ regarding LDL size [86]. In agreement with this study, intakes of SFA- and n-6 PUFA-rich diets (~ 41% E fat) for 6 wk., both diets supplemented with EPA and DHA, displayed no significant differences regarding LDL particle size concentrations [97].

### The effects of saturated fatty acids on LDL particle size

Small LDL concentrations decreased after following a lower-carbohydrate, low-saturated fat diet (38% E fat, 8% E SFA, 21% E MUFA, and 31% E CHO) for 3 wk. compared to a lower-carbohydrate, high-saturated fat diet (38% E fat, 15% E SFA, 15% E MUFA, and 31% E CHO). Intake of the lower-carbohydrate, low-saturated fat diet also decreased medium LDL concentrations in comparison to the lower-carbohydrate, high-saturated fat diet and the baseline diet (38% E fat, 15% E SFA, 15% E MUFA, and 50% E CHO). Both lower-carbohydrate diets were rich in beef protein, whereas the baseline diet was not. No significant differences were observed between diets regarding large and very small LDL concentrations [79]. In a similar study [93], the consumption of a low-carbohydrate, high-saturated fat diet (38% E fat, 15% E SFA, 15% E MUFA, and 31% E CHO) for 3 wk. increased large, medium, and small LDL mass concentrations compared to a low-carbohydrate, low-saturated fat diet (38% E fat, 8% E SFA, 21% E MUFA, and 31% E CHO). There were no significant changes for very small LDL concentrations, which coincide with the previous investigation. Another study reported that the inclusion of various protein sources (red meat, white meat, or vegetable) – along with a high SFA (~ 14% E) intake – increased large LDL particle concentrations compared to the low SFA (~ 7% E) diet after 4 wk.; however, no significant differences were found for small- and medium-sized LDL subclasses [99].

### The effects of saturated and monounsaturated fatty acids on LDL particle size

The intake of a moderate-MUFA diet (39.7% E fat, 12.1% E SFA, and 15.1% E MUFA) for 16 wk. increased the percentage of large LDL particles compared to the SFA reference diet (39.8% E fat, 15.4% E SFA, and 12.5% E MUFA); however, there were no significant differences in the proportions of the LDL subfractions between the moderate-MUFA and high-MUFA (37.1% E, 9.7% E SFA, and 16.6% E MUFA) diets [82]. Following a low-fat diet (28.7% E fat and 7.2% E SFA) and a high-fat diet (40.2% E fat and 9.9% E SFA), both rich in MUFA (13.9% E and 19.8% E, respectively), decreased the size of the major LDL fraction compared to the wash-in SFA-rich diet (40.8% E fat, 18.1% E SFA, and 13.1% E MUFA) after 4 wk. No significant differences, however, were found between the MUFA-rich diets [92]. Interestingly, there were increases in small LDL particle numbers with the consumption of a lower-fat diet (24% E fat, 7% E SFA, 11% E MUFA, and 59% E CHO) or a moderate-fat diet (34% E fat, 6% E SFA, 17% E MUFA, and 49% E CHO) for 5 wk. each in comparison to an average American diet (34% E fat, 13% E SFA, 12% E MUFA, and 51% E CHO). There was also a significant increase in small LDL particle numbers with the lower-fat diet versus the moderate-fat diet. Moreover, intakes of the lower-fat and higher-fat diets reduced large LDL particle numbers compared with the average American diet, which was higher in saturated fat [95].

### The effects of polyunsaturated fatty acids on LDL particle size

It was demonstrated by Dias et al. [96] that intake of an n-6 PUFA diet (38.6% E fat) decreased very large, medium-large, and small LDL particle concentrations compared with a SFA diet (38.8% E fat) after 10 d (both diets were supplemented with EPA and DHA). In agreement with these results, large, medium, and small LDL particle concentrations decreased by following an experimental diet which contained 6.4% E higher PUFA and 6.5% E lower SFA in comparison to the control diet after 8 wk. [98].

### The effects of oils on LDL particles size

The consumption of a sunflower seed oil diet (13.3% E PUFA) increased LDL size compared to an olive oil diet (21.6% E MUFA) after 3 wk. on each diet [81]. In another study, it was reported that the consumption of an olive oil diet increased the number of larger and medium-sized LDL subfractions compared to the rapeseed oil and sunflower oil diets (all 35% E fat) after 3 wk. The olive oil diet also increased the number of medium-sized and sdLDL subfractions compared with the rapeseed oil diet. No significant differences were observed, however, regarding the number of the smallest, dense LDL particles [27]. In contrast to these findings, the intakes of refined olive oil, rapeseed oil, or sunflower oil diets (all ~ 38% E fat) for 4 wk. did not differ significantly regarding LDL size; however, these three diets significantly reduced LDL size compared to a baseline diet rich in SFA (19% E) [85].

### The effects of very-low carbohydrate and ketogenic diets on LDL particle size

The intake of a ketogenic diet (61% E fat and 8% E CHO) increased LDL peak particle diameter (after 3 wk) and LDL-1 percentage (after 3 and 6 wk) compared to a habitual diet (25% E fat and 59% E CHO). Additionally, all initial pattern A subjects stayed pattern A following the ketogenic diet, whereas most initial pattern B participants changed to pattern A following the ketogenic diet [33]. In another investigation, the consumption of a ketogenic diet (77% E fat and 4% E CHO) for 4 wk. increased both sdLDL and large, buoyant LDL particles [100]. There were no significant differences in percentages or concentrations of LDL subclasses in participants following a very-low CHO diet (60% E fat and 10% E CHO) or a low-fat diet (19% E fat and 62% E CHO) for 4 wk. each; however, 3 of 10 individuals with pattern B exhibited larger peak LDL size after the very-low CHO diet [89].

## Discussion

This review sought to summarize intervention studies that explored the effects of fat intake on LDL size in healthy individuals. A summary of the reviewed studies will be presented in this section. The mechanisms of LDL particle formation – along with factors that influence LDL particle production – will be briefly discussed. Additionally, recommendations to decrease the risk factors for CVD will be suggested. Lastly, the major findings and concluding remarks will be covered.

### A summary of the reviewed studies on the effects of fat consumption on LDL particle size

The consumption of high-fat (~ 32 to 46% E) diets increased large, buoyant LDL particles and/or decreased sdLDL particles compared to low-fat (~ 10 to 25% E) diets [71, 77, 80, 84, 91, 94]. About one-third of pattern A subjects switched to pattern B by following low-fat diets [77, 84]. The intakes of higher fat (~ 33 to 41% E) diets with a variety of individual fatty acid compositions resulted in no significant differences in LDL particle size [78, 83, 86, 88, 90, 97]. In the context of a lower-carbohydrate (31% E) diet, the consumption of low-saturated fat (38% E fat and 8% E SFA) or high-saturated fat (38% E fat and 15% E SFA) diets produced mixed results on LDL subclasses [79, 93]. In other studies, high SFA (~ 13 to 19% E) intakes increased large and/or decreased small LDL particle size, numbers, mass, and/or concentrations compared to low SFA (~ 6 to 11% E) intakes, with total fat consumption of ~ 24 to 46% E in the respective diets [71, 85, 92, 95, 99]. The consumption of PUFA-rich diets decreased large, medium, and small LDL concentrations versus diets higher in SFA [96, 98]. The inclusion of diets containing olive oil, sunflower oil, or rapeseed oil produced inconsistent outcomes regarding LDL size, perhaps due to the differing ingredients, such as vitamins and phytochemicals [27, 81, 85]. There were inconsistent results in LDL size after adhering to ketogenic or very-low carbohydrate diets [33, 89, 100]. Lastly, males displayed higher concentrations of sdLDL particles compared to females [26].

Regarding individual fatty acids, myristic acid (2.3% E) and palmitic acid (9% E) increased large LDL particle mass, with myristic acid also decreasing sdLDL mass [71]. There were reductions in very large, medium-large, and small LDL particle concentrations after following a diet rich in linoleic acid compared to a diet high in SFA [96]. In contrast, there were no significant changes in LDL particle mean size after consuming diets rich in lauric acid (10.6% E), palmitic acid (13.2% E), or oleic acid (19% E) [83], or diets low in linoleic acid (3% E linoleic acid and 0.4% E alpha-linolenic acid), high in alpha-linolenic acid (7.1% E linoleic acid and 1.1% E alpha-linolenic acid), or the control provision (7.3% E linoleic acid and 0.4% E alpha-linolenic acid) [78]. Additionally, there were no significant differences in LDL particle size and subclass concentrations after intakes of diets rich in stearic acid (7.7% E), oleic acid (13.1% E), or linoleic acid (9.3% E) [90].

### The mechanisms regarding the production of LDL particle size

**Fig. 2:**
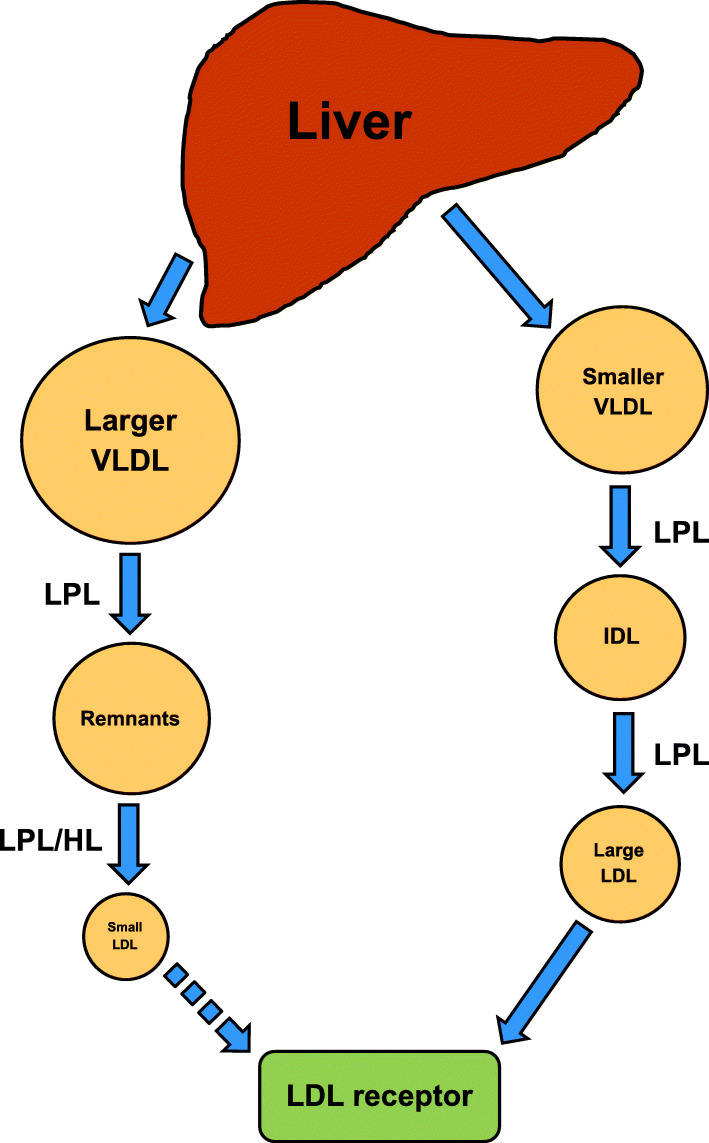
The production of LDL particles. HL, hepatic lipase; IDL, intermediate-density lipoprotein; LDL, low-density lipoprotein; LPL, lipoprotein lipase; VLDL, very-low-density lipoprotein

In a detailed review by Berneis and Krauss [101], it was illustrated that there is “metabolic channeling within the VLDL-IDL-LDL delipidation cascade” in which pathways produce different intermediate-density lipoprotein (IDL) and LDL particles from various precursor TG-rich lipoproteins, such as VLDL. For example, VLDL particles are produced in the liver and the TG in VLDL particles are hydrolyzed by lipoprotein lipase (LPL) in various tissues, thereby generating IDL. The IDL-TG particles are further hydrolyzed, which produce LDL particles. The cholesteryl ester transfer protein (CETP) exchanges the cholesteryl esters in LDL for TG in VLDL. This is followed by hydrolysis of TG in LDL particles by hepatic lipase (HL), which generates sdLDL particles [102]. These sdLDL particles are formed due to “prolonged residence” in the bloodstream – primarily from VLDL particles – and display reduced binding to the LDL receptor [28, 31, 32] (Fig. 2). The CETP, additionally, participates in a similar exchange of lipids between VLDL and HDL particles, which results in HDL that are low in cholesteryl esters [102, 103].

There is a “strong” relationship between plasma TG and sdLDL whereby increased TG concentrations are associated with smaller LDL size. Decreased HDL cholesterol concentrations are also correlated with smaller LDL particles [17]. Very large VLDL particles typically generate lower quantities of LDL as their “delipidation” likely stops in the VLDL or IDL density range which may be removed from plasma or remain in circulation. It has been suggested that there is a relationship between large VLDL particles and sdLDL, as certain individuals convert VLDL to IDL, and eventually sdLDL particles [25, 101]. Interestingly, the intake of a whole food, high-carbohydrate diet increases [TG] [67]. The metabolic reactions that generate sdLDL also generate “abnormalities” in VLDL and HDL subfractions that likely increase CVD risk [34].

### The effects of fat consumption on LDL particle size

The consumption of very-low fat (< 8% E) diets, and consequently, high-carbohydrate diets, reduces lipase activity, such as LPL, whereas a high-fat diet and a higher saturated fat diet increase HL and LPL activities [80, 104]. HL activity has been indicated to be positively correlated with sdLDL particles, whereas LPL activity has been shown to be inversely associated with sdLDL concentrations [80]. However, these enzymes do not seem to be “primary determinants” in generating sdLDL particles. Additionally, HL activity was demonstrated to be inversely correlated with small VLDL and small IDL. These smaller VLDL particles are “more effective” ligands for LDL receptors compared to the larger varieties [101, 105]. Moreover, LPL activity was shown to be positively associated with small IDL and large, buoyant LDL particles. Increased LPL activity stimulated by intake of a high-fat diet may increase the production of large, buoyant LDL particles and small IDL, and increased HL activity may increase the catabolism and clearance of TG-rich lipoprotein remnants [80]. The involvement of CETP in the production of the sdLDL subfraction is controversial, and may depend upon underlying health conditions [101, 103].

According to the reviewed studies [71, 77, 80, 84, 91, 94], the consumption of the high-fat diets increased the large, buoyant LDL class and/or decreased sdLDL particles compared to the low-fat diets. Furthermore, there were increases in LDL particle size following higher SFA intakes compared to lower SFA consumption [71, 85, 92, 95, 99]. As such, the results of these reviewed investigations are in agreement with the proposed mechanisms of fat consumption on LDL particle size. However, additional research is needed regarding the effects of individual fatty acids on LDL particle size.

### The effects of additional factors on LDL particle size

An increased number of sdLDL particles may result from a disorder in the metabolism of TG-rich lipoproteins [101]. Insulin resistance, for example, may cause decreased retention of fatty acids in adipocytes, and thus, increased plasma free fatty acids that are returned to the liver. Hence, this leads to increased synthesis of TG, and therefore, increased production and “residence time” of large, TG-rich VLDL particles during the postprandial period, thereby increasing CVD risk [15, 34, 102, 106].

The more dense LDL subclasses may also be influenced by age, gender, body weight, smoking, exercise, fiber, plant sterols, hormones, postmenopausal status, and oral contraceptive use [80, 107–111]. For example, increased fat deposition is associated with higher amounts of TG, and in turn, reduced LDL particle size [112]. In another example, women have larger LDL particles compared to men, with the sex differences regarding CVD risk narrowing with age [34, 113]. Premenopausal women also display reduced LDL-C and TG concentrations, as well as higher HDL-C levels [114]. There are also genes regulating this metabolic cascade, such as those involving LPL, HL, and CETP; however, there are other candidates – as well as the genetic variants – that may impact this cascade [9, 102].

### LDL particle size as a risk marker for CVD risk

As suggested previously, sdLDL particles are associated with increased TG and apolipoprotein B levels (associated with LDL-C), and decreased HDL-C and apolipoprotein A1 levels (associated with HDL-C) [36]. Additionally, the sdLDL subclass is considered to be more atherogenic, and therefore, a significant risk factor for CVD [12–23]. On the other hand, sdLDL particles may not be an independent risk factor for CVD after other risk factors have been considered, such as total cholesterol, LDL particle number, and LDL-C, HDL-C, and TG concentrations, according to certain studies [24, 34, 115–119]. There are also additional lipid factors to consider, such as lipoprotein(a) [120], the total cholesterol:HDL-C ratio, apolipoproteins, and measuring LDL size, number, and concentrations [24, 99]. Hence, more research is needed to develop strong risk markers for CVD. Moreover, there are non-lipid factors that may increase CVD risk, such as hypertension, endothelial dysfunction, cardiac arrhythmia, oxidative stress, inflammation, thrombosis, the glucose-insulin axis, and the microbiome [10]. Additional CVD risk factors include smoking, obesity, a lack of exercise, a high alcohol consumption [121], and genetic factors [9, 17, 102, 122].

### Dietary recommendations to decrease the risk factors for CVD

The American College of Cardiology/American Heart Association Task Force has recently provided suggestions to reduce CVD risk factors including: consumption of adequate amounts of fruits, vegetables, legumes, whole grains, nuts, lean vegetable or animal protein sources, and fish, as well as reducing the intakes of trans-fatty acids, red meats and processed red meats, refined carbohydrates, cholesterol, sodium, and sugar-sweetened beverages. It is also recommended to replace a % of E from saturated fatty acids with monounsaturated and polyunsaturated fatty acids [121].

The recently published Dietary Guidelines for Americans emphasize using vegetable oils in place of sources high in saturated fat, such as lard, coconut oil, palm kernel oil, palm oil, butter, shortening, high-fat meats, and full-fat dairy products. Additionally, it is suggested to limit the intake of saturated fats to less than 10% E by replacing them with unsaturated fats – especially polyunsaturated fats [123].

According to the World Health Organization, it is recommended that total fat intake should not exceed 30% E, with less than 10% E as saturated fat. Trans-fat consumption should be less than 1% E and replace saturated fats with unsaturated fats (fish, avocado, nuts, and safflower, sunflower, corn, soybean, olive, and canola oils) [124].

In a prospective cohort study, it was noted that a plant-based dietary pattern (fruits, vegetables, beans, and fish) was associated with a reduced risk for heart failure, whereas the intakes of fried food, processed meats, added fats, and sugar-sweetened beverages increased the risk [125]. The PREDIMED investigation reported that a Mediterranean diet supplemented with extra-virgin olive oil or nuts lowered the incidence of cardiovascular events compared to a reduced-fat diet [126]. Further, consumption of a low-fat diet (20% E fat) during a long-term randomized clinical trial, did not reduce the risk for CVD [127]. A report by Kris-Etherton et al. suggested that a moderate intake of fat – 25 to 35% E – should be consumed. It was additionally stated that “extremes in dietary fat should be avoided” [128].

It has been recently proposed that we should focus on whole foods and overall dietary patterns, rather than individual fatty acids [52, 129]. For example, certain populations that consume higher amounts of cholesterol and saturated fat do not display high CHD mortality rates, as they consume more plant foods, as well as monounsaturated and polyunsaturated fatty acids [53]. Moreover, some saturated fatty acid-rich food items have not been demonstrated to increase CVD risk. A possible explanation is due to the food matrix of these particular foods, such as the components of vitamins, minerals, proteins, phospholipids, probiotics, and phytochemicals. Therefore, guidelines should focus on food-based recommendations and overall dietary patterns rather than individual fatty acid recommendations [10, 52].

### Review strengths and limitations

A strength of this narrative review is that it covers human intervention trials on this topic that were published over an extended period of time (1995 to 2021); the reviewed studies provided evidence that fat consumption impacts LDL particle size. Additionally, based on a search of the literature, a review article has not been published in this area. This review, however, has limitations, such as not analyzing risk of bias in the intervention studies and the heterogeneity of the investigations (ages, sex distributions, diets, study durations, and methods).

## Conclusions and future perspectives

**Fig. 3:**
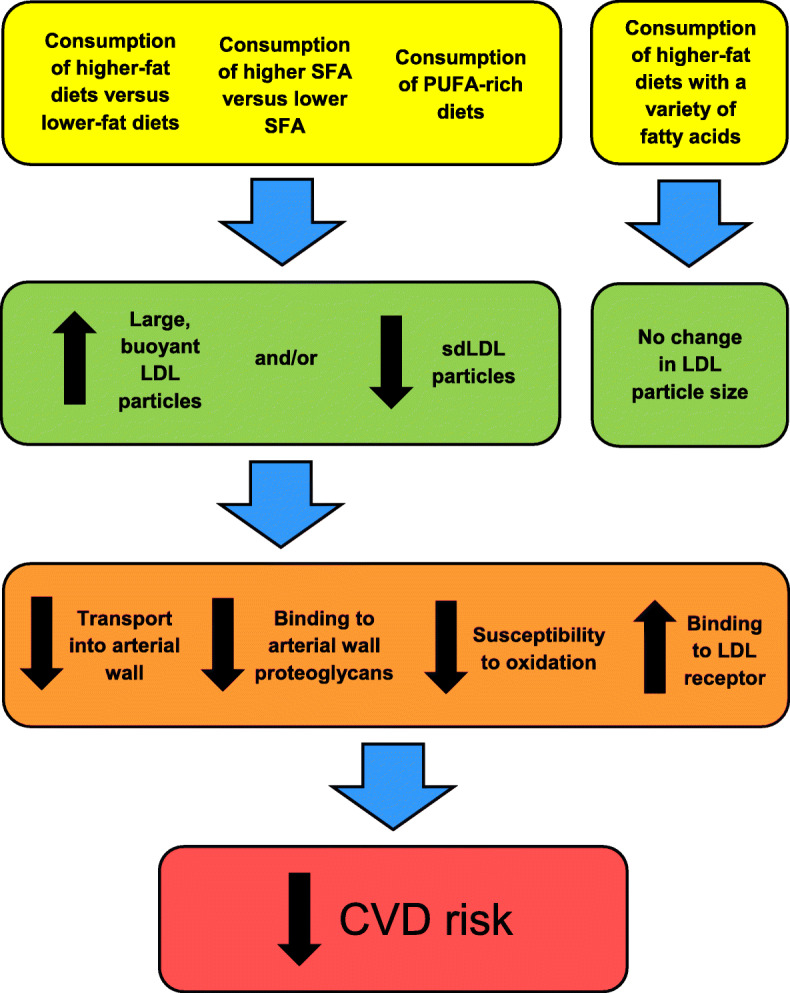
A summary of the major findings from this review. CVD, cardiovascular disease; LDL, low-density lipoprotein; PUFA, polyunsaturated fatty acid; sd, small dense; SFA, saturated fatty acids

The objective of this review was to assess whether fat consumption affects LDL particle size in healthy individuals. Interestingly, it was found that consuming higher fat diets increased large, buoyant LDL and/or decreased sdLDL particles compared to lower fat diets. It was also discovered that higher SFA consumption increased large and/or decreased small LDL particles. In limited studies, intakes of PUFA-rich diets decreased both large and small LDL particles compared to SFA-rich diets. In contrast, the consumption of higher fat diets containing a variety of individual fatty acids did not differ with respect to LDL particle size. Therefore, it appears that the major finding from this review is that LDL particle size is primarily influenced by the consumption of high- and low-fat diets, as well as high and low SFA intakes (Fig. 3). These outcomes emphasize the recommendation to consume adequate amounts of fat to reduce the formation of sdLDL particles, thereby decreasing the risk for CVD. However, more research is needed regarding the effects of individual fatty acids on LDL particle size – in addition to the mechanisms. Overall dietary patterns should also be considered, as food components (macro- and micronutrients, fiber, and phytochemicals) may affect LDL size. It is suggested that further investigations address the effects of dietary patterns (whole versus refined carbohydrates, types and amounts of fat, and plant-based versus meat-based diets) on a variety of cardiovascular disease risk markers – including sdLDL particles. Moreover, it is recommended to ascertain the clinical relevance of the relative and absolute levels, size, number, and peak particle diameter of LDL particles involved with the primary and secondary prevention of CVD.

## Data Availability

Not applicable.

## Abbreviations

CETP: Cholesteryl ester transfer protein
CHD: Coronary heart disease
CVD: Cardiovascular disease
CHO: Carbohydrate
d: days
DHA: Docosahexaenoic acid
E: Energy
EPA: Eicosapentaenoic acid
g: grams
HDL-C: High-density lipoprotein cholesterol
HL: Hepatic lipase
IDL: Intermediate-density lipoproteins
IM: Ion mobility
LDL-C: Low-density lipoprotein cholesterol
LPL: Lipoprotein lipase
MUFA: Monounsaturated fatty acids
NMR: Nuclear magnetic resonance
PAGGE: Polyacrylamide gradient gel electrophoresis
PUFA: Polyunsaturated fatty acids
sdLDL: small dense low-density lipoprotein
SFA: Saturated fatty acids
TG: Triglycerides
UC: Ultracentrifugation
VLDL: Very-low-density lipoproteins
wk.: weeks
